# Crystal structure of 3-*O*-benzyl-4(*R*)-*C*-(1-benzyl-1*H*-1,2,3-triazol-4-yl)-1,2-*O*-iso­propyl­idene-α-d-erythro­furan­ose

**DOI:** 10.1107/S2056989015022434

**Published:** 2015-11-28

**Authors:** Nikita Semjonovs, Vitalijs Rjabovs, Dmitrijs Stepanovs, Maris Turks

**Affiliations:** a Institute of Technology of Organic Chemistry, Faculty of Materials Science and Applied Chemistry, Riga Technical University, P. Valdena 3/7, Riga, LV-1048, Latvia; b Latvian Institute of Organic Synthesis, Str. Aizkraukles 21, Riga, LV 1006, Latvia

**Keywords:** crystal structure, 1,2,3-triazole, click chemistry, carbohydrate triazole conjugate, pseudo-nucleoside

## Abstract

The title compound, C_23_H_25_N_3_O_4_, {systematic name: 1-benzyl-4-[(3a*R*,5*R*,6*R*,6a*R*)-6-benz­yloxy-2,2-di­methyl­tetra­hydro­furo[2,3-*d*][1,3]dioxol-5-yl]-1*H*-1,2,3-triazole}, consists of a substituted 2,2-di­methyl­tetra­hydro­furo[2,3-*d*][1,3]dioxole. The furan­ose ring adopts an envelope conformation close to *C*_3_-*exo*, where the C atom substituted by the benz­yloxy group is the flap. The fused dioxolane ring also adopts an envelope conformation, with the methyl­ene C atom as the flap. In the crystal, mol­ecules are linked by weak C—H⋯O hydrogen bonds, forming zigzag chains along [010].

## Abstract 2

The title compound is a substituted 2,2-di­methyl­tetra­hydro­furo[2,3-*d*][1,3]dioxole. The furan­ose ring adopts an envelope conformation, close to *C*_3_-*exo*, as does the fused dioxolane ring. In the crystal, mol­ecules are linked by weak C—H⋯O hydrogen bonds, forming zigzag chains along [010].

## Chemical context   

**Figure 1:**
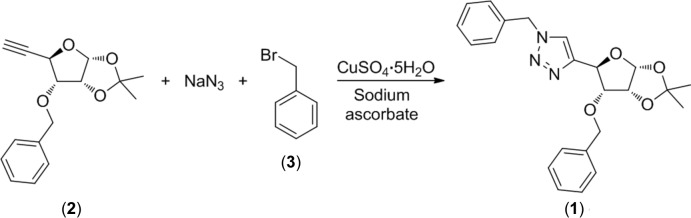
Synthesis of the title compound (**1**).

The title compound, (**1**), was obtained in a one-pot multicomponent click reaction (Rostovtsev *et al.*, 2002 ▸; Kumar *et al.*, 2009 ▸) of alkyne (**2**), sodium azide, and benzyl bromide (**3**), in the presence of copper(II) sulfate and sodium ascorbate in THF solution at 323 K (Fig. 1 ▸). Similar C(4)-linked carbo­hydrate-1,2,3-triazole conjugates have been synthesized under different reaction conditions (Dururgkar *et al.*, 2009 ▸; Kaliappan *et al.*, 2009 ▸; Strakova *et al.*, 2011 ▸). Many carbohydrate-triazole conjugates have been probed as glycosidase inhibitors (Rjabova *et al.*, 2012 ▸), galectin inhibitors (Mackeviča *et al.*, 2014 ▸), and anti­microbial agents (Jana *et al.*, 2014 ▸; Reddy *et al.*, 2014 ▸). Starting alkyne (**2**) and similar carbo­hydrate alkynes have been studied previously as precursors for triazole syntheses (Ciunik & Jarosz, 1998 ▸; Jarosz, 1988 ▸; Rjabovs *et al.*, 2015 ▸; Strakova *et al.*, 2011 ▸).
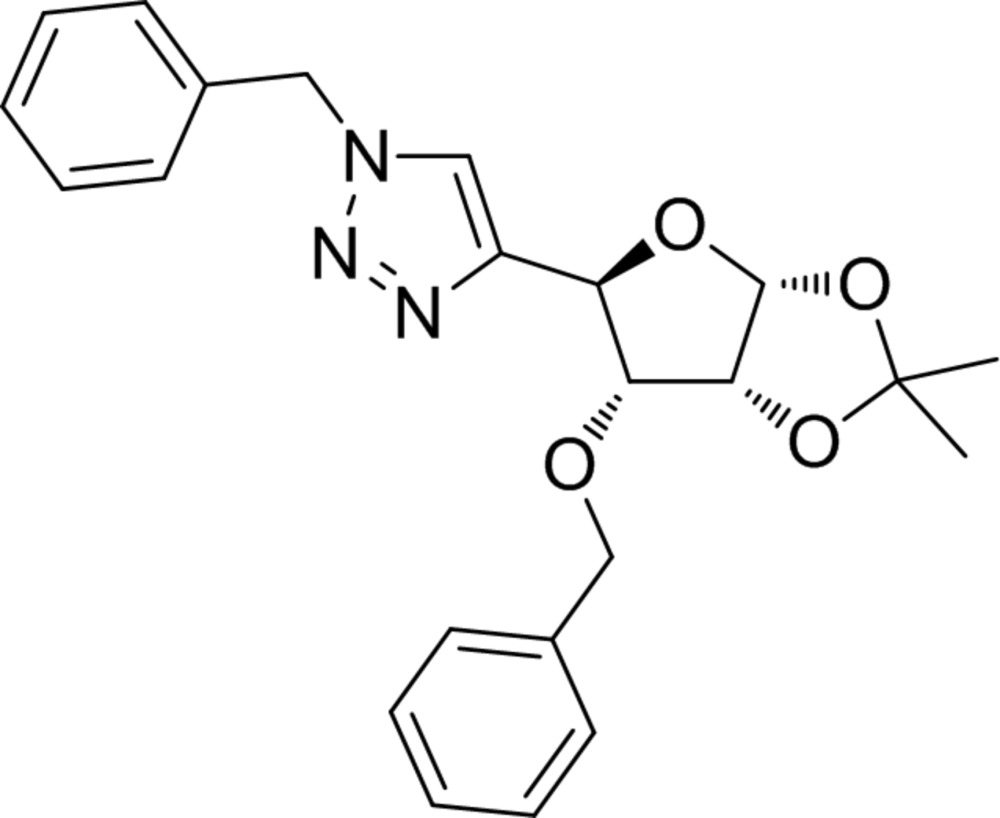


## Structural commentary   

**Figure 2:**
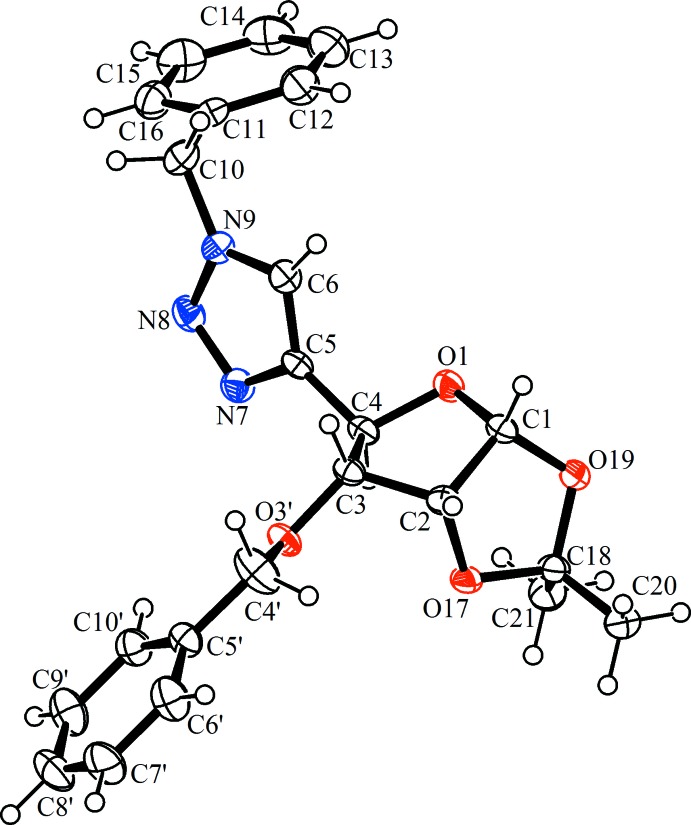
The mol­ecular structure of compound (**1**), showing the atom labelling. Displacement ellipsoids are drawn at the 50% probability level.

The title compound, Fig. 2 ▸, consists of a tetra­hydro­furan core fused with a dioxolane ring, and substituted with benzyl and (1-benz­yl)-1*H*-1,2,3-triazol-4-yl moieties. The furan­ose ring adopts an envelope conformation close to *C*_3_-*exo*, where atom C3 deviates from the mean plane through atoms O1/C1/C2/C4 by 0.577 (4) Å. The fused dioxolane ring also adopts an envelope conformation, where atom C2 deviates from the mean plane through the four near planar atoms (O17/C18/O19/C1) by 0.364 (4) Å. The dihedral angle between the mean planes of the fragments of these rings is 69.3 (1)°.

## Supra­molecular features   

In the crystal, weak C—H⋯O hydrogen bonds (Table 1 ▸) link the mol­ecules, forming zigzag chains along the *b*-axis direction. There are no other significant inter­molecular inter­actions present.

## Database survey   

A search of the Cambridge Structural Database (Version 5.36; Groom & Allen, 2014 ▸) for substituted 3a,5,6,6a-tetra­hydro­furo[2,3-*d*][1,3]dioxoles gave 485 hits (excluding organomet­allics). Three of them are triazoles: (4*R*)-4-(2-allyl-2*H*-1,2,3-triazol-4-yl)-1,2-*O*-iso­propyl­idene-l-threose (LOHTIM; Jenkinson *et al.*, 2008 ▸) and 5-({5-[6-(benz­yloxy)-2,2-di­methyl­tetra­hydro­furo[2,3-*d*][1,3]dioxol-5-yl]-1*H*-1,2,3-triazol-1-yl}meth­yl)-2,2-di­methyl­tetra­hydro­furo[2,3-*d*][1,3]dioxol-6-ol (DOPVAH01 and DOPVEL01, two stereoisomers; Kayet *et al.*, 2014 ▸).

## Synthesis and crystallization   

The synthesis of the title compound is illustrated in Fig. 1 ▸. Sodium azide (98 mg, 1.5 mmol, 3 eq.) was added to a solution of alkyne (**2**) (140 mg, 0.5 mmol, 1 eq.) in THF (10 ml). The mixture was cooled to 273 K and benzyl bromide (**3**) (70 µl, 0.6 mmol, 1.2 eq.) was added. After 20 min solutions of copper(II) sulfate penta­hydrate (12 mg, 10 mol%) in water (0.5 ml) and sodium ascorbate (20 mg, 20 mol%) in water (0.5 ml) were added and the resulting reaction mixture was warmed to 323 K. After 3 h the solvent was evaporated under reduced pressure, the residue was dissolved in EtOAc (20 ml). The organic layer was washed with a saturated aqueous solution of NaHCO_3_ (3 × 5 ml) and brine (3 × 5 ml), dried over Na_2_SO_4_, filtered and evaporated. The solid residue was purified by column chromatography on silica gel eluting with hexa­nes/EtOAc giving a white crystalline solid (yield: 132 mg, 65%; m.p. 430-431 K). Colourless plate-like crystals were obtained by slow evaporation of a di­chloro­methane solution at ambient temperature.

Spectroscopic data: IR (KBr, cm^−1^): 3125, 3085, 2985, 2895, 1495, 1455, 1385, 1370, 1230, 1145, 1100, 1075, 1040, 995. ^1^H NMR (CDCl_3_, 300 MHz): δ 7.37 (*m*, 4H), 7.28–7.16 (*m*, 6H), 5.83 (*d*, *J* = 3.6 Hz, 1H), 5.54 (*d*, AB syst., *J* = 14.8 Hz, 1H), 5.47 (*d*, AB syst., *J* = 14.8 Hz, 1H), 5.13 (*d*, *J* = 9.0 Hz, 1H), 4.64 (*m*, 2H), 4.55 (*d*, AB syst., *J* = 12.2 Hz, 1H), 4.25 (*dd*, *J* = 8.0, 4.0 Hz, 1H), 1.63 (*s*, 3H), 1.37 (*s*, 3H). ^13^C NMR (CDCl_3_, 75 MHz): ^13^C NMR (75 MHz, CDCl_3_) δ 145.12, 137.64, 134.54, 129.28, 128.96, 128.43, 128.27, 128.12, 127.98, 123.26, 113.14, 103.98, 81.43, 77.93, 72.59, 72.57, 54.31, 26.92, 26.54.

## Refinement   

Crystal data, data collection and structure refinement details are summarized in Table 2 ▸. The C-bound H atoms were positioned geometrically and refined as riding on their parent atoms: C—H = 0.93–0.98Å with *U*_iso_(H) = 1.5*U*_eq_(C) for methyl H atoms and 1.2*U*_eq_(C) for other H atoms. Reflection (0,0,2) whose intensity was affected by the beam-stop was removed from the final refinement. In the final cycles of refinement, in the absence of significant anomalous scattering effects, Friedel pairs were merged and Δ*f*′′ set to zero.

## Supplementary Material

Crystal structure: contains datablock(s) I. DOI: 10.1107/S2056989015022434/su5245sup1.cif

Structure factors: contains datablock(s) I. DOI: 10.1107/S2056989015022434/su5245Isup2.hkl

CCDC reference: 1438541

Additional supporting information:  crystallographic information; 3D view; checkCIF report

## Figures and Tables

**Table 1:** Hydrogen-bond geometry (Å, °)

*D*—H⋯*A*	*D*—H	H⋯*A*	*D*⋯*A*	*D*—H⋯*A*
C21—H21*C*⋯O19^i^	0.96	2.53	3.285 (3)	136

Symmetry code: (i) 
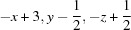
.

**Table 2:** Experimental details

Crystal data	
Chemical formula	C_23_H_25_N_3_O_4_
*M*_r_	407.46
Crystal system, space group	Orthorhombic, *P*2_1_2_1_2_1_
Temperature (K)	173
*a*, *b*, *c* (Å)	9.5276 (2), 10.0030 (2), 21.9495 (7)
*V* (Å^3^)	2091.89 (9)
*Z*	4
Radiation type	Mo *K*α
μ (mm^−1^)	0.09
Crystal size (mm)	0.31 × 0.17 × 0.12

Data collection	
Diffractometer	Nonius KappaCCD
Absorption correction	–
No. of measured, independent and observed [*I* > 2σ(*I*)] reflections	5878, 3423, 1983
*R*_int_	0.070
(sin θ/λ)_max_ (Å^−1^)	0.705

Refinement	
*R*[*F* ^2^ > 2σ(*F* ^2^)], *wR*(*F* ^2^), *S*	0.053, 0.106, 1.02
No. of reflections	3423
No. of parameters	273
H-atom treatment	H-atom parameters constrained
Δρ_max_, Δρ_min_ (e Å^−3^)	0.19, −0.20

Computer programs: *KappaCCD Server Software* (Nonius, 1997 ▸), *DENZO* and *SCALEPACK* (Otwinowski & Minor, 1997 ▸), *SIR2011* (Burla *et al.*, 2012 ▸), *ORTEP-3 for Windows* (Farrugia, 2012 ▸), *SHELXL97* (Sheldrick, 2008 ▸), *PLATON* (Spek, 2009 ▸) and *publCIF* (Westrip, 2010 ▸).

## supplementary crystallographic information

### Crystal data

**Table:**

C_23_H_25_N_3_O_4_	*D*_x_ = 1.294 Mg m^−^^3^
*M**_r_* = 407.46	Mo *K*α radiation, λ = 0.71073 Å
Orthorhombic, *P*2_1_2_1_2_1_	Cell parameters from 11742 reflections
*a* = 9.5276 (2) Å	θ = 1.0–30.0°
*b* = 10.0030 (2) Å	µ = 0.09 mm^−^^1^
*c* = 21.9495 (7) Å	*T* = 173 K
*V* = 2091.89 (9) Å^3^	Plate, colourless
*Z* = 4	0.31 × 0.17 × 0.12 mm
*F*(000) = 864	

### Data collection

**Table:**

Nonius KappaCCD diffractometer	1983 reflections with *I* > 2σ(*I*)
Radiation source: fine-focus sealed tube	*R*_int_ = 0.070
Graphite monochromator	θ_max_ = 30.1°, θ_min_ = 2.2°
φ and ω scan	*h* = −13→13
5878 measured reflections	*k* = −14→14
3423 independent reflections	*l* = −30→30

### Refinement

**Table:**

Refinement on *F*^2^	Primary atom site location: structure-invariant direct methods
Least-squares matrix: full	Secondary atom site location: difference Fourier map
*R*[*F*^2^ > 2σ(*F*^2^)] = 0.053	Hydrogen site location: inferred from neighbouring sites
*wR*(*F*^2^) = 0.106	H-atom parameters constrained
*S* = 1.02	*w* = 1/[σ^2^(*F*_o_^2^) + (0.0425*P*)^2^] where *P* = (*F*_o_^2^ + 2*F*_c_^2^)/3
3423 reflections	(Δ/σ)_max_ < 0.001
273 parameters	Δρ_max_ = 0.19 e Å^−^^3^
0 restraints	Δρ_min_ = −0.20 e Å^−^^3^

### Special details

**Table:**

Geometry. All e.s.d.'s (except the e.s.d. in the dihedral angle between two l.s. planes) are estimated using the full covariance matrix. The cell e.s.d.'s are taken into account individually in the estimation of e.s.d.'s in distances, angles and torsion angles; correlations between e.s.d.'s in cell parameters are only used when they are defined by crystal symmetry. An approximate (isotropic) treatment of cell e.s.d.'s is used for estimating e.s.d.'s involving l.s. planes.
Refinement. Refinement of *F*^2^ against ALL reflections. The weighted *R*-factor *wR* and goodness of fit *S* are based on *F*^2^, conventional *R*-factors *R* are based on *F*, with *F* set to zero for negative *F*^2^. The threshold expression of *F*^2^ > σ(*F*^2^) is used only for calculating *R*-factors(gt) *etc*. and is not relevant to the choice of reflections for refinement. *R*-factors based on *F*^2^ are statistically about twice as large as those based on *F*, and *R*- factors based on ALL data will be even larger.

### Fractional atomic coordinates and isotropic or equivalent isotropic displacement parameters (Å^2^)

**Table:**

	*x*	*y*	*z*	*U*_iso_*/*U*_eq_	
O1	1.16295 (19)	0.88874 (17)	0.19794 (8)	0.0303 (4)	
C1	1.2698 (3)	0.9325 (2)	0.23846 (11)	0.0257 (6)	
H1	1.2802	1.0299	0.2367	0.031*	
C2	1.2262 (3)	0.8879 (2)	0.30203 (12)	0.0264 (6)	
H2	1.2484	0.9535	0.3338	0.032*	
C3	1.0704 (3)	0.8622 (2)	0.29521 (11)	0.0249 (6)	
H3	1.0190	0.9468	0.2983	0.030*	
C4	1.0612 (3)	0.8090 (2)	0.23003 (11)	0.0255 (6)	
H4	1.0914	0.7153	0.2295	0.031*	
C5	0.9254 (3)	0.8207 (2)	0.19804 (11)	0.0247 (6)	
C6	0.8620 (3)	0.9299 (3)	0.17250 (11)	0.0281 (7)	
H6	0.8943	1.0176	0.1724	0.034*	
N7	0.8440 (3)	0.7120 (2)	0.18755 (10)	0.0330 (6)	
N8	0.7314 (3)	0.7502 (2)	0.15626 (10)	0.0340 (6)	
N9	0.7435 (2)	0.8834 (2)	0.14768 (9)	0.0281 (5)	
C10	0.6401 (3)	0.9530 (3)	0.11021 (12)	0.0347 (7)	
H10A	0.6524	1.0487	0.1150	0.042*	
H10B	0.5466	0.9304	0.1243	0.042*	
C11	0.6530 (3)	0.9172 (2)	0.04351 (12)	0.0278 (6)	
C12	0.7666 (4)	0.9615 (3)	0.00985 (13)	0.0424 (8)	
H12	0.8340	1.0160	0.0278	0.051*	
C13	0.7797 (4)	0.9242 (3)	−0.05091 (14)	0.0509 (9)	
H13	0.8563	0.9536	−0.0735	0.061*	
C14	0.6808 (4)	0.8446 (3)	−0.07767 (14)	0.0507 (9)	
H14	0.6908	0.8191	−0.1182	0.061*	
C15	0.5672 (4)	0.8026 (3)	−0.04496 (15)	0.0511 (9)	
H15	0.4987	0.7502	−0.0635	0.061*	
C16	0.5537 (3)	0.8380 (3)	0.01595 (14)	0.0402 (8)	
H16	0.4770	0.8078	0.0382	0.048*	
O17	1.29867 (19)	0.76405 (16)	0.30941 (8)	0.0286 (5)	
C18	1.4170 (3)	0.7600 (2)	0.26879 (11)	0.0278 (6)	
O19	1.3983 (2)	0.86916 (16)	0.22727 (8)	0.0308 (5)	
C20	1.5517 (3)	0.7802 (3)	0.30359 (13)	0.0360 (7)	
H20A	1.5477	0.8636	0.3252	0.054*	
H20B	1.6292	0.7816	0.2757	0.054*	
H20C	1.5639	0.7084	0.3321	0.054*	
C21	1.4123 (3)	0.6299 (2)	0.23368 (12)	0.0364 (7)	
H21A	1.4858	0.6291	0.2038	0.055*	
H21B	1.3230	0.6216	0.2137	0.055*	
H21C	1.4250	0.5564	0.2613	0.055*	
O3'	1.0121 (2)	0.76803 (16)	0.33605 (8)	0.0283 (4)	
C4'	0.9967 (4)	0.8185 (3)	0.39562 (13)	0.0497 (9)	
H4'1	0.9405	0.8992	0.3945	0.060*	
H4'2	1.0883	0.8413	0.4119	0.060*	
C5'	0.9281 (3)	0.7180 (2)	0.43610 (12)	0.0303 (7)	
C6'	0.9439 (4)	0.7281 (3)	0.49853 (14)	0.0446 (8)	
H6'	0.9965	0.7976	0.5150	0.054*	
C7'	0.8820 (4)	0.6357 (3)	0.53642 (13)	0.0495 (9)	
H7'	0.8924	0.6437	0.5784	0.059*	
C8'	0.8054 (4)	0.5322 (3)	0.51301 (15)	0.0500 (9)	
H8'	0.7648	0.4692	0.5388	0.060*	
C9'	0.7889 (4)	0.5223 (3)	0.45101 (15)	0.0480 (9)	
H9'	0.7360	0.4528	0.4348	0.058*	
C10'	0.8498 (3)	0.6139 (3)	0.41277 (12)	0.0360 (7)	
H10'	0.8383	0.6058	0.3709	0.043*	

### Atomic displacement parameters (Å^2^)

**Table:**

	*U*^11^	*U*^22^	*U*^33^	*U*^12^	*U*^13^	*U*^23^
O1	0.0293 (11)	0.0383 (10)	0.0232 (9)	−0.0053 (9)	−0.0004 (9)	0.0065 (8)
C1	0.0310 (17)	0.0218 (14)	0.0244 (14)	−0.0016 (13)	0.0007 (13)	0.0025 (11)
C2	0.0315 (16)	0.0208 (13)	0.0267 (14)	0.0026 (12)	−0.0007 (13)	−0.0005 (11)
C3	0.0319 (16)	0.0216 (12)	0.0211 (13)	0.0002 (13)	0.0030 (13)	−0.0002 (10)
C4	0.0318 (17)	0.0212 (13)	0.0235 (13)	0.0007 (13)	0.0046 (13)	0.0038 (10)
C5	0.0325 (16)	0.0231 (12)	0.0185 (13)	−0.0036 (13)	0.0028 (13)	0.0020 (11)
C6	0.0304 (17)	0.0298 (14)	0.0242 (14)	−0.0049 (13)	−0.0024 (13)	−0.0029 (11)
N7	0.0321 (14)	0.0315 (13)	0.0354 (14)	−0.0074 (12)	−0.0046 (12)	0.0026 (10)
N8	0.0346 (14)	0.0320 (13)	0.0354 (13)	−0.0103 (12)	0.0012 (12)	0.0013 (11)
N9	0.0278 (14)	0.0283 (13)	0.0281 (12)	0.0003 (11)	−0.0023 (11)	−0.0025 (10)
C10	0.0305 (17)	0.0389 (15)	0.0347 (16)	0.0069 (15)	−0.0020 (14)	−0.0042 (13)
C11	0.0285 (16)	0.0281 (14)	0.0268 (14)	0.0061 (13)	−0.0068 (13)	−0.0018 (12)
C12	0.042 (2)	0.0478 (19)	0.0371 (18)	−0.0066 (17)	−0.0070 (16)	−0.0003 (14)
C13	0.053 (2)	0.061 (2)	0.0384 (19)	0.0017 (19)	0.0041 (18)	0.0066 (17)
C14	0.071 (3)	0.053 (2)	0.0280 (17)	0.0107 (19)	−0.0061 (19)	−0.0054 (15)
C15	0.061 (3)	0.0497 (18)	0.043 (2)	−0.0042 (19)	−0.016 (2)	−0.0110 (15)
C16	0.0359 (19)	0.0394 (17)	0.0452 (19)	−0.0010 (15)	−0.0034 (17)	−0.0013 (14)
O17	0.0303 (11)	0.0232 (9)	0.0324 (10)	0.0064 (8)	0.0054 (9)	0.0073 (8)
C18	0.0317 (16)	0.0252 (13)	0.0266 (14)	0.0049 (13)	0.0040 (13)	0.0039 (11)
O19	0.0279 (11)	0.0299 (9)	0.0346 (10)	0.0030 (9)	0.0061 (9)	0.0087 (8)
C20	0.0371 (18)	0.0359 (16)	0.0350 (16)	0.0024 (14)	−0.0021 (15)	0.0045 (13)
C21	0.0406 (19)	0.0271 (14)	0.0415 (17)	0.0033 (15)	0.0032 (16)	−0.0038 (12)
O3'	0.0390 (11)	0.0255 (9)	0.0203 (9)	−0.0023 (9)	0.0070 (8)	0.0003 (8)
C4'	0.080 (3)	0.0394 (17)	0.0298 (17)	−0.0144 (19)	0.0176 (18)	−0.0088 (13)
C5'	0.0356 (18)	0.0296 (14)	0.0258 (15)	−0.0002 (14)	0.0070 (13)	−0.0038 (11)
C6'	0.050 (2)	0.0518 (19)	0.0317 (17)	−0.0111 (18)	0.0060 (16)	−0.0077 (14)
C7'	0.060 (2)	0.064 (2)	0.0240 (16)	−0.003 (2)	0.0109 (17)	0.0030 (15)
C8'	0.061 (3)	0.0463 (19)	0.043 (2)	−0.0046 (18)	0.0200 (18)	0.0107 (15)
C9'	0.053 (2)	0.0410 (17)	0.050 (2)	−0.0119 (17)	0.0146 (19)	−0.0063 (16)
C10'	0.0406 (18)	0.0404 (17)	0.0270 (15)	−0.0024 (16)	0.0031 (14)	−0.0011 (13)

### Geometric parameters (Å, º)

**Table:**

O1—C1	1.421 (3)	C14—H14	0.9300
O1—C4	1.439 (3)	C15—C16	1.389 (4)
C1—O19	1.400 (3)	C15—H15	0.9300
C1—C2	1.523 (3)	C16—H16	0.9300
C1—H1	0.9800	O17—C18	1.438 (3)
C2—O17	1.428 (3)	C18—O19	1.433 (3)
C2—C3	1.514 (4)	C18—C20	1.507 (4)
C2—H2	0.9800	C18—C21	1.513 (3)
C3—O3'	1.414 (3)	C20—H20A	0.9600
C3—C4	1.529 (3)	C20—H20B	0.9600
C3—H3	0.9800	C20—H20C	0.9600
C4—C5	1.477 (4)	C21—H21A	0.9600
C4—H4	0.9800	C21—H21B	0.9600
C5—N7	1.355 (3)	C21—H21C	0.9600
C5—C6	1.369 (3)	O3'—C4'	1.409 (3)
C6—N9	1.337 (3)	C4'—C5'	1.492 (4)
C6—H6	0.9300	C4'—H4'1	0.9700
N7—N8	1.330 (3)	C4'—H4'2	0.9700
N8—N9	1.351 (3)	C5'—C10'	1.379 (4)
N9—C10	1.460 (3)	C5'—C6'	1.382 (4)
C10—C11	1.513 (3)	C6'—C7'	1.376 (4)
C10—H10A	0.9700	C6'—H6'	0.9300
C10—H10B	0.9700	C7'—C8'	1.367 (4)
C11—C16	1.374 (4)	C7'—H7'	0.9300
C11—C12	1.383 (4)	C8'—C9'	1.373 (4)
C12—C13	1.391 (4)	C8'—H8'	0.9300
C12—H12	0.9300	C9'—C10'	1.371 (4)
C13—C14	1.366 (4)	C9'—H9'	0.9300
C13—H13	0.9300	C10'—H10'	0.9300
C14—C15	1.365 (5)		
			
C1—O1—C4	110.29 (18)	C13—C14—H14	120.0
O19—C1—O1	112.16 (19)	C14—C15—C16	120.0 (3)
O19—C1—C2	105.47 (19)	C14—C15—H15	120.0
O1—C1—C2	106.7 (2)	C16—C15—H15	120.0
O19—C1—H1	110.8	C11—C16—C15	120.5 (3)
O1—C1—H1	110.8	C11—C16—H16	119.8
C2—C1—H1	110.8	C15—C16—H16	119.8
O17—C2—C3	109.7 (2)	C2—O17—C18	109.46 (17)
O17—C2—C1	103.1 (2)	O19—C18—O17	105.97 (19)
C3—C2—C1	103.1 (2)	O19—C18—C20	109.0 (2)
O17—C2—H2	113.3	O17—C18—C20	110.5 (2)
C3—C2—H2	113.3	O19—C18—C21	109.1 (2)
C1—C2—H2	113.3	O17—C18—C21	108.4 (2)
O3'—C3—C2	115.8 (2)	C20—C18—C21	113.5 (2)
O3'—C3—C4	109.82 (19)	C1—O19—C18	110.00 (19)
C2—C3—C4	102.0 (2)	C18—C20—H20A	109.5
O3'—C3—H3	109.6	C18—C20—H20B	109.5
C2—C3—H3	109.6	H20A—C20—H20B	109.5
C4—C3—H3	109.6	C18—C20—H20C	109.5
O1—C4—C5	108.25 (19)	H20A—C20—H20C	109.5
O1—C4—C3	103.10 (19)	H20B—C20—H20C	109.5
C5—C4—C3	117.9 (2)	C18—C21—H21A	109.5
O1—C4—H4	109.1	C18—C21—H21B	109.5
C5—C4—H4	109.1	H21A—C21—H21B	109.5
C3—C4—H4	109.1	C18—C21—H21C	109.5
N7—C5—C6	108.5 (2)	H21A—C21—H21C	109.5
N7—C5—C4	121.3 (2)	H21B—C21—H21C	109.5
C6—C5—C4	130.2 (2)	C4'—O3'—C3	113.00 (19)
N9—C6—C5	105.2 (2)	O3'—C4'—C5'	110.9 (2)
N9—C6—H6	127.4	O3'—C4'—H4'1	109.5
C5—C6—H6	127.4	C5'—C4'—H4'1	109.5
N8—N7—C5	108.6 (2)	O3'—C4'—H4'2	109.5
N7—N8—N9	106.6 (2)	C5'—C4'—H4'2	109.5
C6—N9—N8	111.0 (2)	H4'1—C4'—H4'2	108.0
C6—N9—C10	129.3 (2)	C10'—C5'—C6'	118.8 (3)
N8—N9—C10	119.5 (2)	C10'—C5'—C4'	121.6 (2)
N9—C10—C11	112.1 (2)	C6'—C5'—C4'	119.5 (3)
N9—C10—H10A	109.2	C7'—C6'—C5'	120.2 (3)
C11—C10—H10A	109.2	C7'—C6'—H6'	119.9
N9—C10—H10B	109.2	C5'—C6'—H6'	119.9
C11—C10—H10B	109.2	C8'—C7'—C6'	120.7 (3)
H10A—C10—H10B	107.9	C8'—C7'—H7'	119.7
C16—C11—C12	119.2 (3)	C6'—C7'—H7'	119.7
C16—C11—C10	120.5 (3)	C7'—C8'—C9'	119.2 (3)
C12—C11—C10	120.3 (3)	C7'—C8'—H8'	120.4
C11—C12—C13	119.8 (3)	C9'—C8'—H8'	120.4
C11—C12—H12	120.1	C10'—C9'—C8'	120.7 (3)
C13—C12—H12	120.1	C10'—C9'—H9'	119.7
C14—C13—C12	120.4 (3)	C8'—C9'—H9'	119.7
C14—C13—H13	119.8	C9'—C10'—C5'	120.4 (3)
C12—C13—H13	119.8	C9'—C10'—H10'	119.8
C15—C14—C13	120.0 (3)	C5'—C10'—H10'	119.8
C15—C14—H14	120.0		
			
C4—O1—C1—O19	109.7 (2)	N9—C10—C11—C12	−70.7 (3)
C4—O1—C1—C2	−5.3 (3)	C16—C11—C12—C13	−0.8 (4)
O19—C1—C2—O17	−24.2 (2)	C10—C11—C12—C13	177.9 (3)
O1—C1—C2—O17	95.2 (2)	C11—C12—C13—C14	0.3 (5)
O19—C1—C2—C3	−138.4 (2)	C12—C13—C14—C15	0.8 (5)
O1—C1—C2—C3	−19.0 (3)	C13—C14—C15—C16	−1.6 (5)
O17—C2—C3—O3'	44.2 (3)	C12—C11—C16—C15	0.1 (4)
C1—C2—C3—O3'	153.50 (19)	C10—C11—C16—C15	−178.6 (3)
O17—C2—C3—C4	−75.0 (2)	C14—C15—C16—C11	1.1 (5)
C1—C2—C3—C4	34.3 (2)	C3—C2—O17—C18	131.7 (2)
C1—O1—C4—C5	152.8 (2)	C1—C2—O17—C18	22.4 (2)
C1—O1—C4—C3	27.2 (3)	C2—O17—C18—O19	−12.5 (2)
O3'—C3—C4—O1	−161.15 (19)	C2—O17—C18—C20	105.5 (2)
C2—C3—C4—O1	−37.8 (2)	C2—O17—C18—C21	−129.5 (2)
O3'—C3—C4—C5	79.7 (3)	O1—C1—O19—C18	−98.2 (2)
C2—C3—C4—C5	−156.9 (2)	C2—C1—O19—C18	17.6 (3)
O1—C4—C5—N7	136.0 (2)	O17—C18—O19—C1	−4.0 (3)
C3—C4—C5—N7	−107.6 (3)	C20—C18—O19—C1	−123.0 (2)
O1—C4—C5—C6	−41.0 (4)	C21—C18—O19—C1	112.5 (2)
C3—C4—C5—C6	75.3 (3)	C2—C3—O3'—C4'	74.5 (3)
N7—C5—C6—N9	0.4 (3)	C4—C3—O3'—C4'	−170.7 (3)
C4—C5—C6—N9	177.7 (2)	C3—O3'—C4'—C5'	177.0 (2)
C6—C5—N7—N8	−0.1 (3)	O3'—C4'—C5'—C10'	−20.4 (4)
C4—C5—N7—N8	−177.7 (2)	O3'—C4'—C5'—C6'	159.0 (3)
C5—N7—N8—N9	−0.3 (3)	C10'—C5'—C6'—C7'	−0.2 (5)
C5—C6—N9—N8	−0.5 (3)	C4'—C5'—C6'—C7'	−179.6 (3)
C5—C6—N9—C10	−174.5 (2)	C5'—C6'—C7'—C8'	0.6 (5)
N7—N8—N9—C6	0.5 (3)	C6'—C7'—C8'—C9'	−0.9 (5)
N7—N8—N9—C10	175.2 (2)	C7'—C8'—C9'—C10'	0.8 (5)
C6—N9—C10—C11	103.7 (3)	C8'—C9'—C10'—C5'	−0.3 (5)
N8—N9—C10—C11	−69.9 (3)	C6'—C5'—C10'—C9'	0.0 (5)
N9—C10—C11—C16	108.0 (3)	C4'—C5'—C10'—C9'	179.4 (3)

### Hydrogen-bond geometry (Å, º)

**Table:**

*D*—H···*A*	*D*—H	H···*A*	*D*···*A*	*D*—H···*A*
C21—H21*C*···O19^i^	0.96	2.53	3.285 (3)	136

Symmetry code: (i) −*x*+3, *y*−1/2, −*z*+1/2.
